# Near-Wellbore Fracture Diagnosis via Strain Decoupling from Integrated In-Well LF-DAS and DTS Data

**DOI:** 10.3390/s26061813

**Published:** 2026-03-13

**Authors:** Jiayi Song, Weibo Sui, Huan Guo, Jiwen Li

**Affiliations:** College of Petroleum Engineering, China University of Petroleum (Beijing), Beijing 102249, China; 2022310158@student.cup.edu.cn (J.S.); 2024310190@student.cup.edu.cn (H.G.); 2023210330@student.cup.edu.cn (J.L.)

**Keywords:** in-well LF-DAS, fracture diagnosis, distributed acoustic sensing

## Abstract

The low-frequency distributed acoustic sensing (LF-DAS) data acquired through fiber-optic cables cemented behind the fracturing well casing can dynamically capture the hydraulic fracturing process. After removing the thermal effect, the LF-DAS data can reveal the strain evolution induced by the initiation of hydraulic fractures. This paper presented an improved strain–temperature decoupling method for LF-DAS measurements based on joint LF-DAS/distributed temperature sensing (DTS) monitoring. The decoupling method was based on strain change and temperature change pre-processed from the raw DAS and DTS data to avoid the enhancement of DTS data noise. The moving window function method and the image processing parameter cosine similarity was introduced to cope with the differences in temporal and spatial resolution between LF-DAS and DTS data. The region significantly affected by temperature change could be identified automatically and the mechanical strain change could be extracted. The tensile strain response generally reached a local peak at perforation clusters and increased significantly at those with dominant fracture fluid inflow. By analyzing the evolution of strain profile during fracturing, the effectiveness of multi-cluster fracture initiation and fracture temporary plugging could be evaluated.

## 1. Introduction

Distributed fiber-optic sensing (DFOS) has emerged as a key technology for hydraulic fracturing monitoring and plays a vital role in unconventional reservoir development due to its distributed measurement capability and real-time visualization advantage [1,2]. With high sampling frequencies, distributed acoustic sensing (DAS) enables monitoring of transient downhole and reservoir events throughout the fracturing process. The application of DAS in fracturing monitoring can be categorized into two primary scenarios, cross-well monitoring and in-well monitoring, based on the relationship between the monitoring and fracturing wells. The geometry of hydraulic fractures can be inverted from the far-field strain obtained by cross-well LF-DAS strain monitoring [3,4,5]. Information obtained from in-well DAS monitoring directly reflects fluid flow in the wellbore or in the near-wellbore zone [6,7]. In-well DAS monitoring can accurately locate downhole events and identify the cross-flow [8]. There have been some theoretical methods for interpreting flow profiling based on in-well DAS monitoring [9,10,11].

Distributed strain sensing based on Rayleigh frequency shift (DSS-RFS) is the first type of fiber-optic sensing adopted for near-wellbore strain monitoring in producing wells. DSS-RFS enables high-spatial-resolution measurement of strain profiles along the fractured horizontal well after shut-in, providing critical data for evaluating the effectiveness of hydraulic fractures [12,13]. In recent years, researchers have integrated DTS with DSS-RFS to remove temperature-induced strain components from DSS-RFS measurements during fracturing monitoring [14,15]. Generally, DSS-RFS samples every few minutes while LF-DAS has a sampling frequency typically between 0.1 and 2 Hz [12,15,16,17,18,19,20]. Compared with DSS-RFS, LF-DAS can better capture valuable transient information during fracturing, since hydraulic fracture initiation and propagation are extremely rapid processes.

LF-DAS measurements reflect temperature changes with high accuracy [21,22,23]. Based on the in-well LF-DAS and concurrent DTS measurements, Leggett decoupled the temperature and mechanical strain components of LF-DAS measurements to observe the mechanical strain change profile along the wellbore during fracturing [24]. His work provides critical insights for assessing the dynamic effectiveness of hydraulic fractures. However, it has not yet been applied to more specific scenarios such as near-wellbore fracture characterization and temporary fracture plugging effectiveness evaluation.

In this paper, LF-DAS and DTS data were recorded simultaneously during segmented multi-cluster fracturing in a horizontal well. Mechanical strain profiles were then obtained after applying an improved decoupling method (coded by Python Version 3.11.4) to remove the temperature-affected component of the LF-DAS signal. By analyzing the evolution of the mechanical strain profile during fracturing, the effectiveness of temporary fracture plugging was assessed and the strain response features in near-wellbore zone during fracturing were identified.

## 2. Mechanism of In-Well LF-DAS Response During Fracturing

Phase-based DAS instruments can measure the phase of backscattered laser pulses travelling in the optical fiber and record the data as optical phase or phase rate. When coherent light propagates through a fiber segment of gauge length, the optical phase can be expressed as [25]

(1) $$\phi =\frac{4\pi nL_{G}}{\lambda }$$

where ϕ is the optical phase in radians, λ is the wavelength, m; *n* is the refractive index, dimensionless; *L*_G_ is the gauge length, m.

Assume the wavelength of light remains constant during monitoring (λ = 1550 nm), the optical phase change can be expressed as [26]

(2) $$\Delta \phi =\phi \left(\frac{\Delta L}{L}+\frac{\Delta n}{n}\right)$$

where *L* represents the length of the optical fiber, m.

When the optical fiber cable is deployed and cemented behind the casing, the optical phase response monitored during fracturing is influenced by both reservoir deformation and temperature change induced by fracturing fluid injection. Temperature change affects fiber responses through thermal expansion and the thermo-optic effect [27]. Thermal expansion alters the fiber length (Equation (3)), while the thermo-optic effect modifies the refractive index of light in the fiber (Equation (4)),

(3) $$\Delta L_{T}=\alpha L\Delta T$$

(4) $$\Delta n_{T}=\zeta_{T}n\Delta T$$

where *α* represent thermal expansion coefficient, °C^−1^; *ζ*_T_ is composite temperature coefficient, °C^−1^; *T* is temperature, °C.

The axial strain on the fiber also induces changes in the refractive index [27],

(5) $$\Delta n_{\varepsilon }=\gamma n\varepsilon$$

where *ε* is the axial strain, *ε* = Δ*L*/*L*, dimensionless; *γ* is the strain-optical coefficient, dimensionless.

The effects of axial strain and temperature on fiber length and refractive index can be written as

(6) $$\frac{\Delta L}{L}=\frac{\Delta L_{M}+\Delta L_{T}}{L}=\varepsilon_{M}+\alpha \Delta T$$

(7) $$\frac{\Delta n}{n}=\gamma \frac{\Delta L}{L}+\zeta_{T}\Delta T$$

where subscripts M and T denote mechanical and thermal effects, respectively.

Substituting Equations (1), (6) and (7) into Equation (2) and simplifying yields

(8) $$\Delta \phi =C_{\varepsilon }\varepsilon_{M}+C_{T}\Delta T$$

where $C_{\varepsilon }=\frac{4\pi nL_{G}}{\lambda }\left(1+\gamma \right)$ and $C_{T}=\frac{4\pi nL_{G}}{\lambda }\left(\zeta_{T}+\gamma \alpha +\alpha \right)$.

According to the technical documentation provided by the service company, the measured optical phase change can be converted into equivalent strain ($\varepsilon_{E}$) using Equation (9),

(9) $$\Delta \phi =C_{\varepsilon }\varepsilon_{E}$$

Substituting Equation (9) into Equation (8) gives

(10) $$\varepsilon_{E}=\varepsilon_{M}+C\Delta T$$

where $C=C_{T}/C_{\varepsilon }$. When the thermal effect dominates and mechanical influence is negligible, the equivalent strain simplifies to $\varepsilon_{E}=C\Delta T$.

## 3. LF-DAS and DTS Data Pre-Processing

The DFOS data utilized in this paper were obtained from a multi-stage fractured horizontal well (hereafter referred to as Well A) in a shale reservoir with poorly developed natural fractures. Post-fracturing core analysis indicates that bedding planes in this shale reservoir had little influence on the hydraulic fracture propagation. The fiber-optic cable was cemented behind the casing to enable simultaneous DTS and DAS measurements. The DAS measurements represent the phase rate in radians per second, with a gauge length of 5 m and a spatial sampling interval of 1 m. The DTS recorded temperature at 30-s intervals, with a spatial sampling interval of 0.5 m.

**Figure 1:**
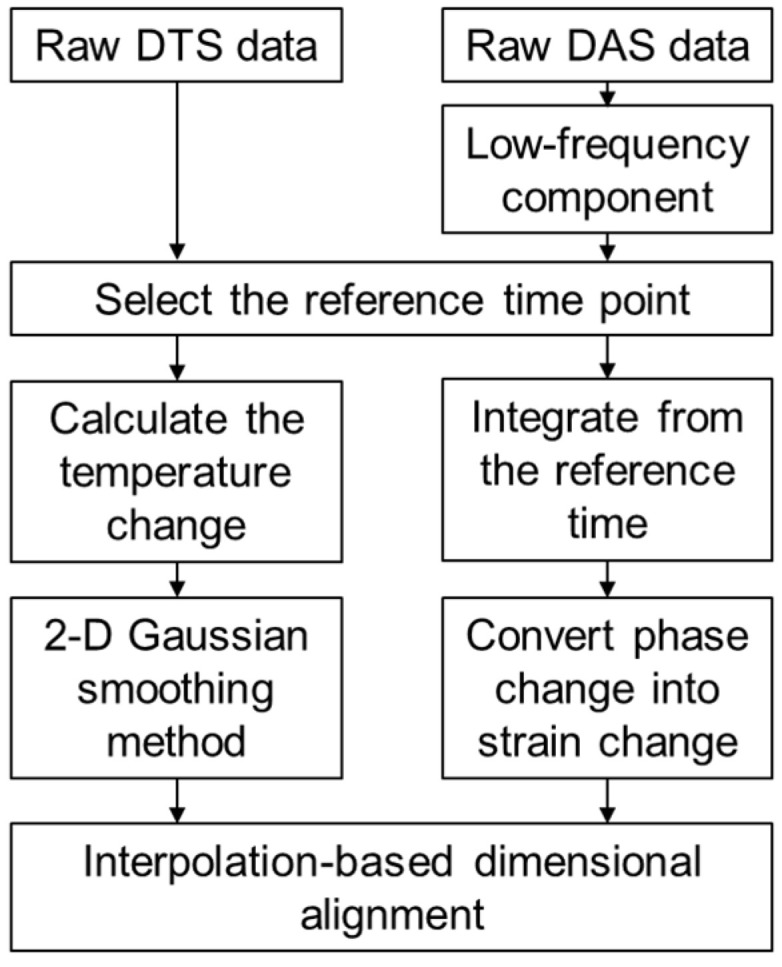
LF-DAS and DTS data pre-processing workflow.

Compared to LF-DAS data, DTS data displays stronger amplitude oscillations that intensify with the length of fiber [28]. This noise cannot be entirely removed using only moving-average or Gaussian smoothing methods. In a previous study [24], the mechanical strain rate was obtained by decoupling the temperature-induced strain component (derived from DTS differential calculations) from the total strain rate measured by LF-DAS. This decoupled mechanical strain rate was then integrated to determine the mechanical strain. The implementation of differential calculations on the DTS data intensifies the original measurement noise, which subsequently appears as recurrent polarity inversions in the temperature change rate visualized by waterfall plots in later analysis. The noise intensity exhibits comparable magnitude to the valuable signal intensity, creating substantial processing challenges. Therefore, we pre-processed the raw DTS and DAS data by converting them into temperature change and strain change parameters to prevent additional amplification of the inherent noise in DTS. A flowchart outlining the LF-DAS and DTS data pre-processing steps is presented in Figure 1.

**Figure 2:**
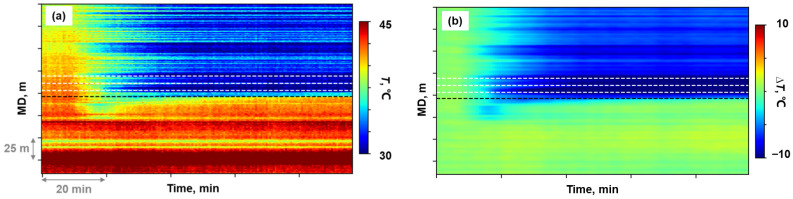

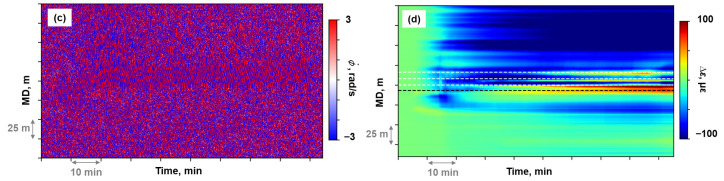
DFOS data waterfall plots before and after pre-processing: (**a**) DTS raw data; (**b**) Processed temperature change; (**c**) DAS raw data; (**d**) Processed equivalent strain change.

The moment in shut-in period measurements prior to stimulation initiation was selected as the time baseline. The temperature change profile along the wellbore was then derived by computing differential value relative to this baseline throughout the fracturing treatment. Subsequently, the temperature change data were processed by first reducing noise using a two-dimensional Gaussian smoothing method with a standard deviation of two [24]. Figure 2a,b illustrate the waterfall plot before and after DTS data pre-processing. The white and black dashed lines in Figure 2 denote the location of the perforation clusters and plug, respectively.

The low-frequency component was extracted from the raw DAS measurements through a digital signal processing method. The raw DAS data were first processed with a low-pass filter. Since the filtered data remained voluminous, the sampling frequency was reduced to 2 Hz through down-sampling. Median filtering was then applied to suppress spike noise, followed by direct current removal to eliminate the background noise. The low-frequency output, originally recorded as phase rate, was subsequently transformed into strain rate through Equation (9). The identical baseline reference time established during DTS data pre-processing was selected to integrate the strain rate data temporally, obtaining strain change along the wellbore over the entire fracturing treatment. Figure 2c,d illustrate the waterfall plot before and after DAS data pre-processing.

Due to the different acquisition frequency and spatial sampling interval of DAS and DTS data, the preprocessed data exhibited dimensional mismatch, which was resolved through interpolation-based dimensional alignment.

## 4. Extraction Method of Mechanical Strain from LF-DAS Data

**Figure 3:**
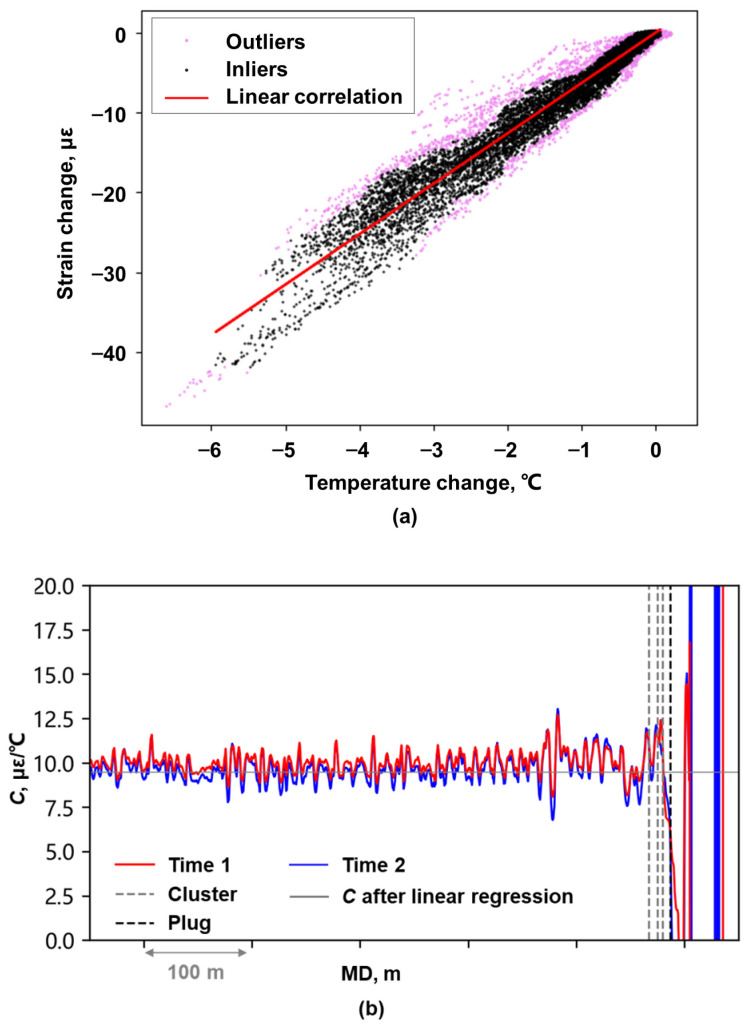
Scaling factor calculation: (**a**) Linear regression of scaling factor *C* after outlier removal; (**b**) Comparison between the depth-specific scaling factor and the scaling factor after linear regression.

The DAS data analyzed in this study contained only the horizontal well section due to storage limitations, preventing subsequent derivation of the scaling factor *C* (Equation (10)) using DAS and DTS data from the vertical well section. Regarding the fracture-swarm identified in shale core samples [29], some researchers [30] suggest it may be associated with cement sheath damage during hydraulic fracturing, potentially enabling fracture initiation in non-perforated well sections adjacent to perforation clusters. Considering both the data characteristics and probable cement sheath integrity compromise during hydraulic fracturing, the scaling factor between temperature change and strain change was calculated using data from non-fractured well section located 50–150 m from the heel-side perforation cluster of the actively fractured stage. The scaling factor between the two sets of data was determined through linear least-squares regression following outlier removal using Local Outline Factor (Figure 3a).

Cosine similarity (CS) was incorporated as a quantitative evaluation parameter in the decoupling method to precisely identify zones where the strain response exhibit temperature correlation. CS measures the similarity between two non-zero vectors, with applications spanning image processing, natural language processing, and machine learning.

**Figure 4:**
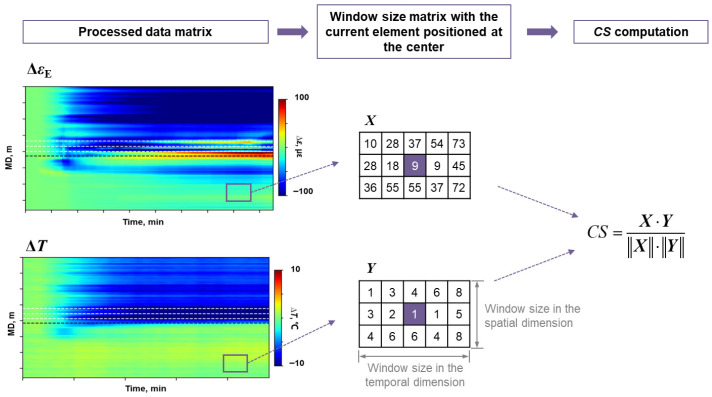
The schematic diagram of CS calculation based on the moving window method.

In this study, CS was used to evaluate the degree of similarity in the spatial direction of 2 non-zero vectors [31]. The CS value varies between −1 and 1, with 1 corresponding to vectors parallel in the same direction and −1 to those parallel in the opposite direction. The closer two vectors are to being parallel in space, the closer the absolute value of their CS value approaches 1. To address the temporal and spatial resolution differences between LF-DAS and DTS measurement, a moving window function was incorporated in the decoupling method. The decoupling process was based on the 2-dimensional window-size data feature instead of single-point data value. The CS calculation based on the moving window method is shown in Figure 4.

During the fracturing process, the horizontal section can be divided into three parts: Section I: the well section far from the actively fractured stage towards the heel side, Section II: the actively fractured stage, and Section III: the section from the plug to the toe side of the horizontal section. Section I is primarily influenced by the cooling effect of the fracturing fluid. Section II is subjected to both the cooling effect and the influence of fracture initiation and propagation. During the ball-drop operation, the temperature change caused by the fracturing fluid can be monitored in Section III. It is also possible to monitor the weak and slow warm-back in the well section towards the toe side in Section III. A CS value less than 0 indicates that the temperature change at the current location is not the dominant factor, or not the sole dominant factor influencing the measured equivalent strain change.

**Figure 5:**
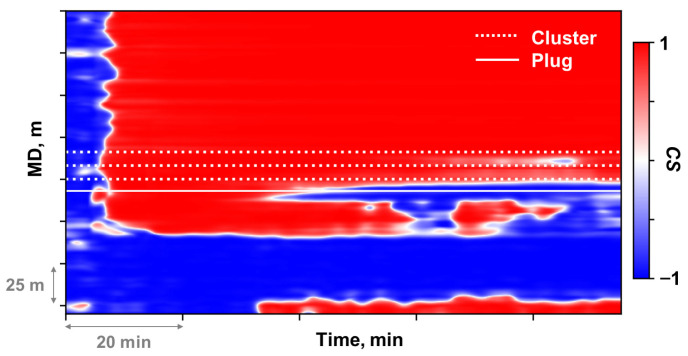
The CS waterfall plot of a fracture stage of Well A during the fracturing.

**Figure 6:**
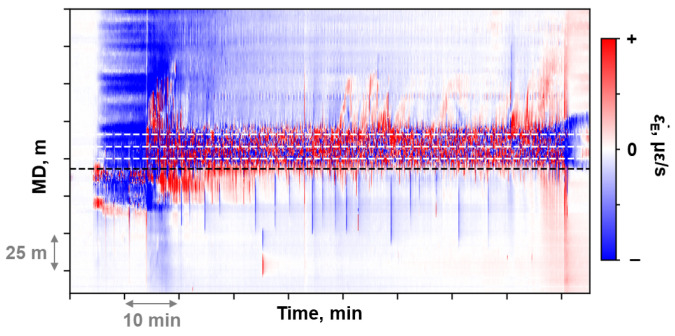
The equivalent strain rate waterfall of a fracture stage.

Theoretically, Section II is expected to exhibit a negative CS. However, during the fracturing process in Well A, the temperature difference between the fracturing fluid and the reservoir was significant. The tensile strain caused by fracture propagation could not completely offset the compressive strain induced by the cooling effect, resulting in a positive CS in Section II (Figure 5). The maximum equivalent strain rate (${\dot{\varepsilon }}_{E\;\max}$) at the current window position was introduced as a secondary discriminator to distinguish Section II from Section I. The equivalent strain rate is an intermediate parameter obtained from DAS data pre-processing. Figure 6 illustrates the equivalent strain rate waterfall plot during the fracturing process of a stage. The white dashed lines in Figure 6 indicate the cluster location, while the black dashed line represents the plug position. From the equivalent strain rate waterfall plot, tensile response caused by fracture initiation and propagation can be observed, whereas Section I typically shows compressive response dominated by the cooling effect.

The above decoupling method can be summarized as Equation (11).

(11) $$\left\{\begin{array}{ll} \Delta \varepsilon_{E} & CS\leq 0,{\dot{\varepsilon }}_{E\max}<0\\ \Delta \varepsilon_{E}-C\Delta T & else \end{array}\right.$$

## 5. Results and Discussion

### 5.1. Decoupling Method Performance

**Figure 7:**
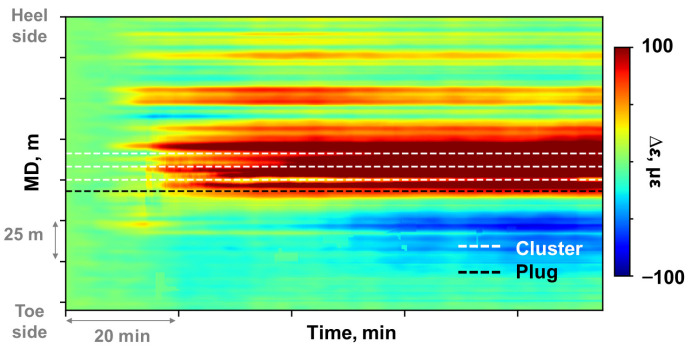
Mechanical strain change waterfall plot after decoupling processing.

The window size applied in this study was 7 m in the spatial dimension and 210 s in the temporal dimension. This window size satisfied the spatial resolution of LF-DAS and met the sampling interval of DTS. Comparing the waterfall plots before (Figure 2d) and after decoupling (Figure 7), the strain change in the non-fractured heel-side well section approached 0 after decoupling, confirming effective suppression of cooling-induced compressive strain. Enhanced tensile strain responses were observed at perforation cluster locations within the actively fractured stage. The compressive strain response observed in the toe-side well section near the bridge plug (Figure 7) may result from bonding degradation at the fiber-cement-formation interfaces.

**Figure 8:**
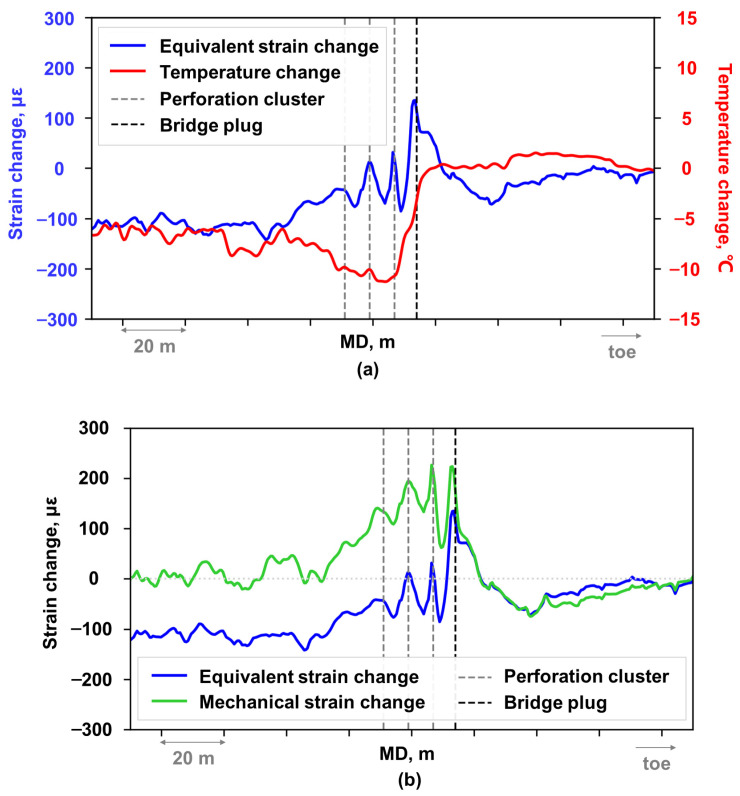
Curves at pumping-end moment: (**a**) Equivalent strain change versus temperature change; (**b**) Equivalent strain change versus mechanical strain change.

A comparison of temperature change and strain change profile along the wellbore during fracturing (Figure 8a) a showed similar trend in the non-fractured well section, whereas a significant difference occurred within the actively fractured well section. The decoupled mechanical strain profile of actively fractured stage exhibited a localized tensile strain peak at or near the cluster location (Figure 8b).

### 5.2. Hydraulic Fracture Effectiveness Evaluation

The propagation of hydraulic fractures induces tensile strain in the behind-casing fiber near the fracture initiation site. Analysis of mechanical strain profiles obtained from the decoupling process, particularly the evolution of tensile strain response, enables assessment of the dynamic effectiveness of hydraulic fracture propagation during fracturing. In this study, it was found that the hydraulic fracture effectiveness evaluation results based on mechanical strain profiles were consistent with those derived from high-frequency DAS waterfall plots.

**Figure 9:**
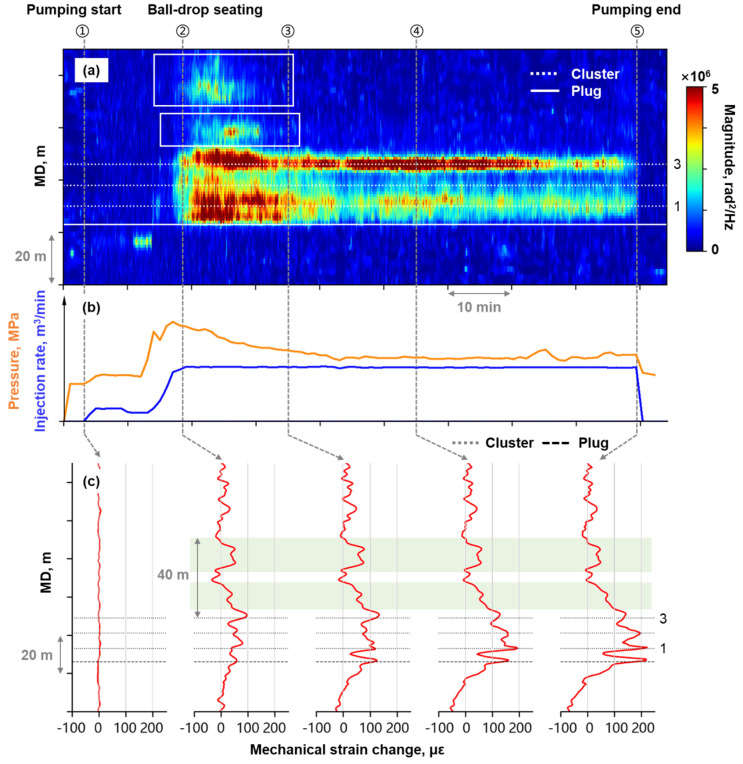
(**a**) High-frequency DAS waterfall plot at Stage A; (**b**) The fracturing treatment curve; (**c**) Timing-slicing plots of mechanical strain profile at Stage A.

Stage A has 3 perforation clusters at 8 m spacing, with a stable injection rate maintained during fracturing. Figure 9a presents the high-frequency DAS waterfall plot for Stage A fracturing. The vertical gray dashed lines in Figure 9 indicate specific timestamps during fracturing. The Arabic number on the right side of Figure 9 indicates the number of the cluster. Figure 9a demonstrates that all three clusters exhibited acoustic signal characteristics of fracturing fluid entry during fracturing.

Figure 9c presents the timing-slicing plots of mechanical strain profile at Stage A, with subplots aligned to the labeled timestamps. After the start of pumping, pronounced tensile strain was detected across the perforated well section. As fracturing progressed, three distinct strain localized peaks developed at the cluster location, exhibiting continuous strain magnitude increase. Therefore, it is concluded that fracture initiation occurred at all three perforation clusters in Stage A, with no fracture closure observed during fracturing despite the potential initiation competition.

Clear acoustic responses were observed in the heel-side non-perforated well section (area marked with white box in Figure 9a) on the high-frequency waterfall plot. Consistent with this observation, the mechanical strain profile showed strong tensile strain development in the corresponding area (green highlighted area in Figure 9c). Tensile strain was observed in the non-perforated well section simultaneously with cluster acoustic signal response, covering a 40 m heel-side area from Cluster 3. The strain remained stable until pumping ended.

Over half of the fracturing stages in this horizontal well exhibited this phenomenon. The studied well is not an isolated case. Haustveit [32] similarly reported a field case that the spatial extent of strong acoustic responses during fracturing significantly exceeded the perforated well section. The observed response may result from fracturing fluid flow both through perforation tunnels into the formation and along the casing-cement interface due to cement sheath damage.

### 5.3. Temporary Plugging Effectiveness Evaluation

**Figure 10:**
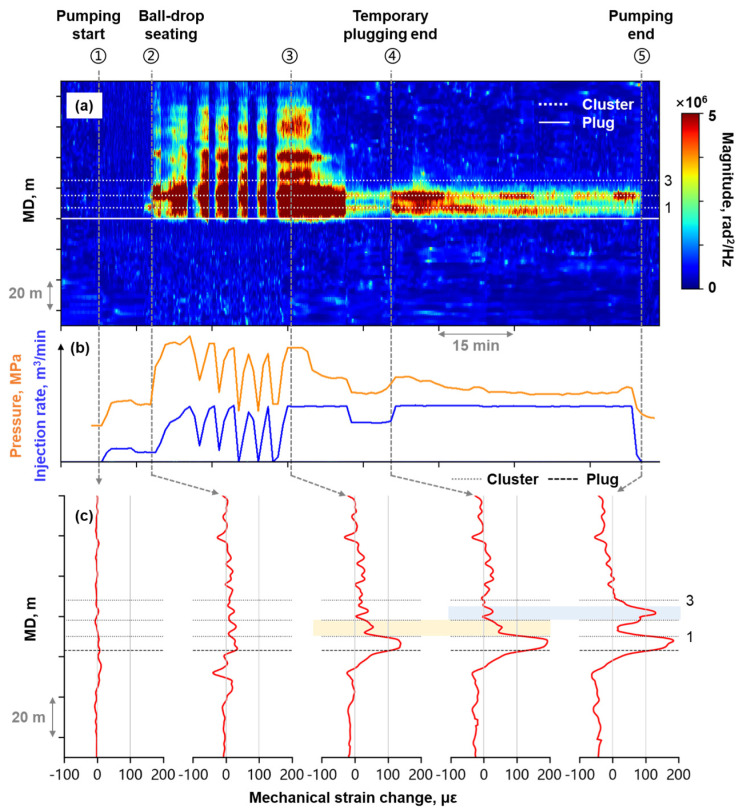
(**a**) High-frequency DAS waterfall plot at Stage B; (**b**) The fracturing treatment curve; (**c**) Timing-slicing plots of mechanical strain profile at Stage B.

Mechanical-strain-based and high-frequency-DAS-derived assessments of temporary plugging performance showed agreement. The high-frequency DAS waterfall plot (Figure 10a) clearly demonstrated preferential fluid entry into the first two perforation clusters during Stage B fracturing, where cluster spacing was maintained at 8–10 m. A single temporary plugging operation (Time ④) was performed during the fracturing of this stage, yet Cluster 3 remained unresponsive after the operation.

While high-frequency DAS waterfall plots suffer from signal overlap due to extensive acoustic coverage, mechanical strain profile timing-slicing plots distinctly show individual cluster response (Figure 10c). In the early stage of fracturing, a localized tensile strain peak was observed near Cluster 1 and Cluster 2, while Cluster 3 exhibited negligible strain. Before temporary plugging operation, a localized tensile strain peak developed between Cluster 1 and 2 (yellow highlighted area in Figure 10c). After temporary plugging operation, this strain diminished while a new peak emerged between Cluster 2 and 3 (blue highlighted area in Figure 10c), with Cluster 3 persistently showing no significant strain response. These findings indicated that the temporary plugging operation improved the fracturing fluid allocation uniformity proximal to Cluster 2, while Cluster 3 remained unstimulated.

In most field applications, high-frequency DAS waterfall plots enable real-time visualization of fracturing fluid allocation in the actively fractured stage. However, effective response may occasionally be absent from high-frequency DAS waterfall plots due to currently unidentified reasons. In such cases, analyzing decoupled mechanical strain profiles can serve as an alternative approach to evaluate both hydraulic fracture effectiveness and temporary plugging performance.

**Figure 11:**
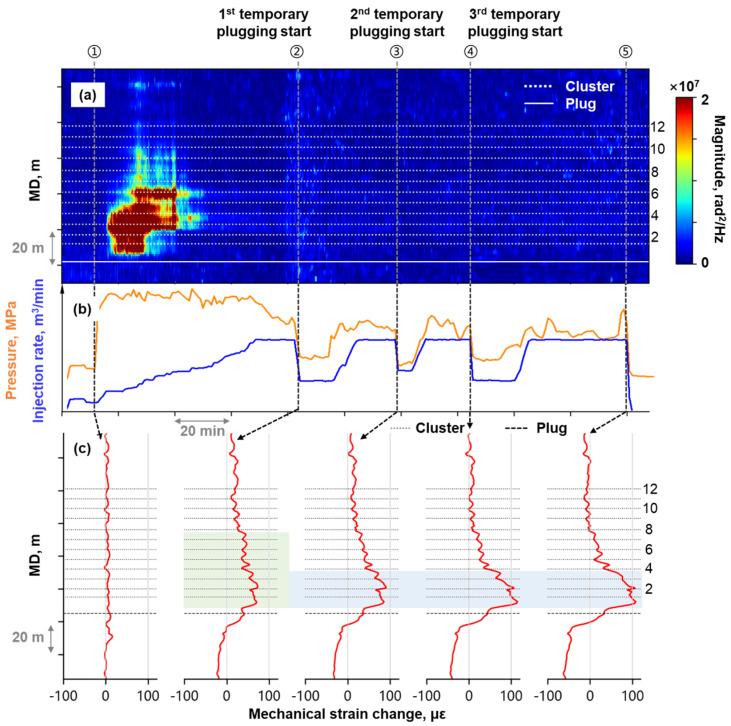
(**a**) High-frequency DAS waterfall plot at Stage C; (**b**) The fracturing treatment curve; (**c**) Timing-slicing plots of mechanical strain profile at Stage C.

**Figure 12:**
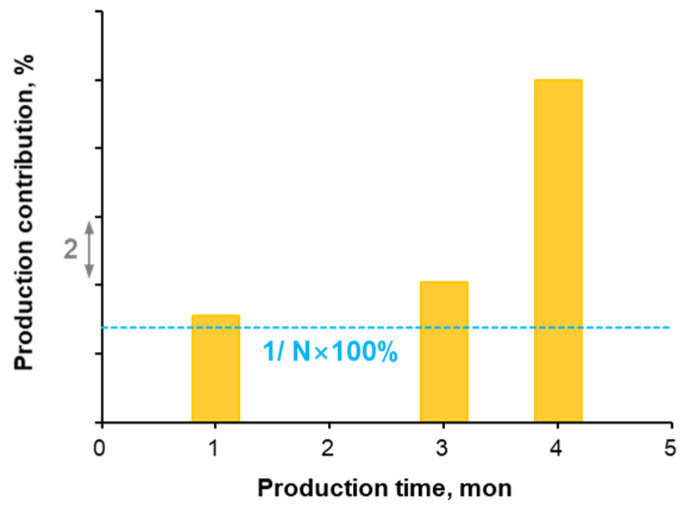
Stage C’s production contribution tracer monitoring result chart.

Stage C was perforated with 12 clusters at 6–8 m spacing, and underwent temporary plugging operations 3 times during fracturing. High-frequency DAS monitoring lost detectable signal after 50 min of fracturing (Figure 11a), eliminating the capability to visually track fracturing fluid allocation or evaluate temporary plugging performance thereafter. However, pressure and injection rate trend showed no anomalies after the acoustic signal loss (Figure 11b), demonstrating uninterrupted fracture propagation. Tracer-based production logging (Figure 12) revealed that Stage C was the primary contributor to production for this well. In Figure 12, *N* represents the total number of fractured stages in Well A. This also confirmed that hydraulic fractures were created effectively during Stage C’ s fracturing process.

Stage C’s mechanical strain profiles (Figure 11c) provided substantially richer fracturing diagnostics compared to the high-frequency DAS waterfall plot. Before the first temporary plugging was implemented (Time ②), tensile strain developed at all clusters, with notably higher strain amplitude observed between Clusters 1 and 7 (green highlighted area in Figure 11c). Following temporary plugging operation, progressive tensile strain accumulation was observed at Clusters 1–3(blue highlighted area in Figure 11c), while other clusters exhibited negligible strain change. Mechanical strain profiling revealed that repeated temporary plugging operations in Stage C resulted in concentrated fluid entry into Cluster 1 to Cluster 3, rather than improving allocation uniformity across all clusters.

**Figure 13:**
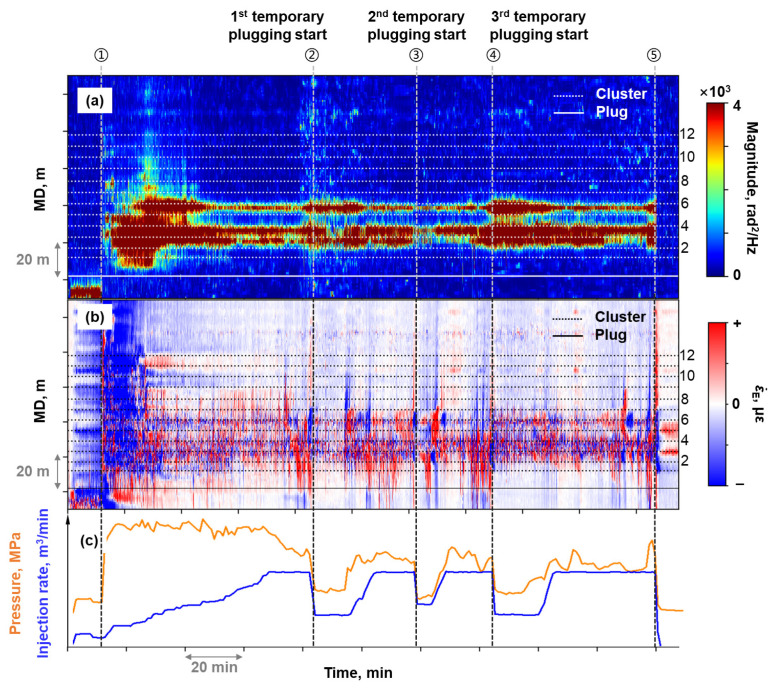
(**a**) 0–100 Hz DAS waterfall plot at Stage C; (**b**) The equivalent strain rate waterfall of Stage C; (**c**) The fracturing treatment curve.

There was no other independent measurement to monitor the fracturing fluid allocation in every cluster. A series of spectral analyses were performed on the raw DAS data of Stage C. Finally, a continuously acoustic response was observed in the 0–100 Hz DAS waterfall plot. Figure 13a is the 0–100 Hz DAS waterfall plot of stage C. The magnitude in this frequency band was significantly lower than that in the 0–5000 Hz range. It could be observed that continuous responses occurred at Clusters 2 to Cluster 4 and Cluster 6 during the fracturing process. Tensile responses were observed at Clusters 1–4 and Cluster 6 in the equivalent strain rate waterfall plot (Figure 13b). The region with observed responses in the 0–100 Hz waterfall plot and the equivalent strain rate waterfall plot partially coincided with the region of tensile strain in the mechanical strain profile.

### 5.4. Limitation of the Method

The method presented in this work has two main limitations. Firstly, debonding of the optical cable from the cement or formation may occur during the fracturing process, causing the raw data measurements to fail to represent the actual physical quantities. Thus, the value of the mechanical strain change after decoupling does not always strictly reflect the strain caused by reservoir deformation. Secondly, the method presented in this study (Equation (11)) paid more attention to the regions exhibiting tensile strain response, which might lead to the neglect of hydraulic fractures that partially close during fracturing.

## 6. Conclusions

In this study, we presented an improved temperature-decoupling method utilizing concurrent DAS/DTS measurements, with successful application to a multi-stage fractured horizontal well in a shale reservoir. Through the post-processing and analysis of the fiber optic data, we obtained the following insights:

(1) The enhanced decoupling method enables effective extraction of mechanical strain from raw DAS measurements.
(2) Fracture propagation and temporary plugging effectiveness assessment derived from mechanical strain profiles show agreement with high-frequency DAS waterfall plot interpretation. However, the decoupled mechanical strain does not always accurately reflect deformation-induced strain from the reservoir, since fiber cable and cement debonding is possible.
(3) Strong acoustic responses were observed in non-perforated heel-side well section on high-frequency DAS waterfall plots, with corresponding tensile strain identified in mechanical strain profiles. This combined evidence suggests fracturing fluid migration through the casing-cement interface due to cement sheath damage.
(4) In-well DAS/DTS monitoring provides enhanced resolution for analyzing cluster-scale fracture dynamics. Where high-frequency DAS waterfall plots prove inconclusive for fluid entry assessment, mechanical strain profile analysis can serve as an alternative to evaluate both hydraulic fracture propagation effectiveness and temporary plugging performance.

## Data Availability

The raw data that support the finding of this study are not publicly available due to commercial restriction.

## Abbreviations

The following abbreviations are used in this manuscript:

DFOS	Distributed fiber optic sensing
DAS	Distributed acoustic sensing
DTS	Distributed temperature sensing
LF-DAS	Low-frequency distributed acoustic sensing
DSS-RFS	Distributed strain sensing based on Rayleigh frequency shift
CS	Cosine similarity
